# Quantitative Genome-Wide Analysis of Yeast Deletion Strain Sensitivities to Oxidative and Chemical Stress

**DOI:** 10.1002/cfg.391

**Published:** 2004-04

**Authors:** Chandra L. Tucker, Stanley Fields

**Affiliations:** 1 Department of Genome Sciences Howard Hughes Medical Institute University of Washington Box 357730 Seattle WA 98195 USA; 2 Department of Medicine Howard Hughes Medical Institute University of Washington Box 357730 Seattle WA 98195 USA; 3 Howard Hughes Medical Institute Depts of Genome Sciences and Medicine University of Washington Box 357730 Seattle WA 98195 USA

## Abstract

Understanding the actions of drugs and toxins in a cell is of critical importance to medicine, yet many of the molecular events involved in chemical resistance are relatively uncharacterized. In order to identify the cellular processes and pathways targeted by chemicals, we took advantage of the haploid *Saccharomyces cerevisiae* deletion strains (Winzeler *et al.,* 1999). Although ~4800 of the strains are viable, the loss of a gene in a pathway affected by a drug can lead to a synthetic lethal effect in which the combination of a deletion and a normally sublethal dose of a chemical results in loss of viability. WE carried out genome-wide screens to determine quantitative sensitivities of the deletion set to four chemicals: hydrogen peroxide, menadione, ibuprofen and mefloquine. Hydrogen peroxide and menadione induce oxidative stress in the cell, whereas ibuprofen and mefloquine are toxic to yeast by unknown mechanisms. Here we report the sensitivities of 659 deletion strains that are sensitive to one or more of these four compounds, including 163 multichemicalsensitive strains, 394 strains specific to hydrogen peroxide and/or menadione, 47 specific to ibuprofen and 55 specific to mefloquine.We correlate these results with data from other large-scale studies to yield novel insights into cellular function.


**Comparative and Functional Genomics**
*Comp Funct Genom* 2004; **5**: 216–224.
Published online in Wiley InterScience (www.interscience.wiley.com). **DOI:** 10.1002/cfg.391

Research Article

### Quantitative genome-wide analysis of yeast deletion strain sensitivities to oxidative and chemical stress

Chandra L. Tucker[1] and Stanley Fields[1,2]*
[1]Department of Genome Sciences, Howard Hughes Medical Institute, University of Washington, Box 357730, Seattle, WA 98195, USA
[2]Department of Medicine, Howard Hughes Medical Institute, University of Washington, Box 357730, Seattle, WA 98195, USA

*Correspondence to:
Stanley Fields, Howard Hughes Medical Institute, Depts of Genome Sciences and Medicine, University of Washington, Box 357730, Seattle, WA 98195, USA.
E-mail: fields@u.washington.edu

Received: 12 August 2003
Revised: 6 January 2004
Accepted: 27 January 2004

### Abstract

**Understanding the actions of drugs and toxins in a cell is of critical importance to medicine, yet many of the molecular events involved in chemical resistance are relatively uncharacterized. In order to identify the cellular processes and pathways targeted by chemicals, we took advantage of the haploid *Saccharomyces cerevisiae* deletion strains (Winzeler *et al.*, 1999). Although ∼4800 of the strains are viable, the loss of a gene in a pathway affected by a drug can lead to a synthetic lethal effect in which the combination of a deletion and a normally sublethal dose of a chemical results in loss of viability. We carried out genome-wide screens to determine quantitative sensitivities of the deletion set to four chemicals: hydrogen peroxide, menadione, ibuprofen and mefloquine. Hydrogen peroxide and menadione induce oxidative stress in the cell, whereas ibuprofen and mefloquine are toxic to yeast by unknown mechanisms. Here we report the sensitivities of 659 deletion strains that are sensitive to one or more of these four compounds, including 163 multichemical-sensitive strains, 394 strains specific to hydrogen peroxide and/or menadione, 47 specific to ibuprofen and 55 specific to mefloquine. We correlate these results with data from other large-scale studies to yield novel insights into cellular function.** **Copyright © 2004 John Wiley & Sons, Ltd.**

**Keywords:** yeast deletion strains; oxidant; stress; sensitivity

### Introduction

The yeast deletion strains, generated by the *Saccharomyces* Genome Deletion Project (Winzeler *et al.*, 1999), are an ordered set of yeast strains in which each open reading frame has been systematically replaced with a kanamycin cassette. Screening these strains for sensitivity to chemical compounds can uncover synthetic lethal effects, in which the combination of a compound and a deletion leads to lethality. Deletion sets were previously screened for sensitivity to compounds including rapamycin, mycophenolic acid, the proteasome inhibitor PS-341, nystatin, methyl methanesulphonate (MMS), wortmannin, amiodarone and gentamycin (Chan *et al.*, 2000; Desmoucelles *et al.*, 2002; Fleming *et al.*, 2002; Giaever *et al.*, 2002; Chang *et al.*, 2002; Hanway *et al.*, 2002; Zewail *et al.*, 2003; SenGupta *et al.*, 2003; Blackburn and Avery, 2003). Although these studies uncovered novel genes, they had several limitations. Sensitivities were generally determined for only a single compound or class, such that strains with sensitivity to unrelated chemicals could not be distinguished from strains with specific sensitivity to a single class of compounds. Often sensitivities were measured on solid plates (typically scored as no growth, poor growth, normal growth), allowing the identification of only 30–100 strains. This focus on the most sensitive strains misses processes that may be more subtly affected by a chemical, which may be ultimately more revealing. These qualitative

Copyright © 2004 John Wiley & Sons, Ltd.

data were also not easily comparable with other genomic data. The use of microarrays that detect 20-mer identifiers that have been PCR-amplified from competitive growth assays of pooled strains generated quantitative data (Fleming *et al.*, 2002; Giaever *et al.*, 2002) but is costly and involves indirect measurements that substitute for growth assays.

With these issues in mind, we carried out quantitative screens of the haploid *MAT*$\alpha$ deletion set in liquid cultures, so as to identify strains with modest but reproducible sensitivity. In addition, we screened with multiple diverse chemicals to compare and contrast the cellular processes targeted. We used two compounds, hydrogen peroxide and menadione, that are of a similar class and have well-characterized targets in both yeast and mammals. These compounds are of special interest as they induce oxidative stress in the cell, which is associated with a range of human diseases. Subsets of deletion strains have previously been screened with oxidants (Higgins *et al.*, 2001; Begley *et al.*, 2002) but a genome-wide screen has not been reported. We also screened with ibuprofen, a compound that has known targets in humans, the cyclooxygenase proteins, but not in yeast, which lack these proteins. The fourth compound we used was mefloquine, an antimalarial agent that is toxic to yeast and has an unknown antimalarial mechanism of action.

### Materials and methods

### Reagents and strains

Haploid yeast deletion strains BY4742 (*MAT*$\alpha$; *his*3$\Delta$1; *leu*2$\Delta$0; *lys*2$\Delta$0; *ura*3$\Delta$0) generated by the *Saccharomyces* Genome Deletion Project (**http://www-sequence.stanford.edu/group/yeast_deletion_project/deletions3.html**) were obtained from Research Genetics (Huntsville, AL). Mefloquine was a gift from Dr William Ellis at the Walter Reed Army Institute of Research. All other chemicals were from Sigma.

### Screening

Titrations were initially carried out in the BY4742 parent strain to identify a concentration of chemical causing ~80–95% of control growth after 20 h. These concentrations, equivalent to 2 mM $H_2O_2$, 67 μM menadione, 50 μM ibuprofen or 235 μM mefloquine, were then used for assays.

Strains were grown individually overnight at 30 °C in YEPD in 96-well plates. Plates were vortexed, then strains were pinned in duplicate using a Biomek 2000 robot (Beckman) into 96-well plates containing complete-synthetic media with no added chemical, followed by plates containing a specific chemical. Plates were briefly vortexed, then incubated at 30 °C for approximately 20 h. Growths were determined by measurement of $OD_{600}$ of non-agitated 96-well plates using a Wallac Victor plate reader.

### Growth calculations

Growth ratios were calculated by dividing the $OD_{600}$ measurement of untreated strains by the measurement of the duplicate chemical-treated strains. An arbitrary constant (the background $OD_{600}$ of 0.033) was included in measurements, so as to avoid the presence of a zero denominator for strains with no growth in the presence of chemicals. Each growth ratio was divided by the median growth ratio of each plate to adjust for plate-to-plate variation in chemical concentration. Ratios for very poor-growing strains ($OD_{600} < 0.1$ for both chemical and untreated plates) were not calculated. Strains with median growth ratios from at least 3 assays of 1.5 or greater (or 2 assays for hydrogen peroxide screens) were rearrayed into new 96-well plates and at least three additional screens with each chemical were carried out. Summary growth ratios reported for sensitive strains (Supplemental Table 2) are equal to the median growth ratios from between 3–9 independent experiments. Strains with a median ratio <1.5 after the first set of screens did not undergo rescreening. Slow-growing strains are those that have an average growth in the absence of chemical that is greater than 2 standard deviations from the median growth of all strains. These are indicated as 'slow-growers' in Supplemental Table 2.

### Data Sets

Raw data and supplemental tables can be downloaded at: **http://depts.washington.edu/sfields/deletion/index.html**.

Copyright © 2004 John Wiley & Sons, Ltd. 

### Results and discussion

### Experimental design

We first assayed growth of the parent strain to determine a concentration of each chemical that resulted in 80–95% cell survival after ∼20 h. We then assayed growth of each deletion strain in the presence or absence of each compound using these concentrations. Ratios were calculated that represent the growth of each strain untreated divided by growth of the strain treated with chemical, such that a higher ratio indicates greater sensitivity. Slow-growing strains tended to yield higher ratios, both because relatively smaller differences in growth have a greater significance in a ratio, and because these already compromised strains may have difficulty dealing with additional chemical stress. Indeed, ∼88% of the 200 poorest-growing strains had a ratio of >1.5 for at least one chemical, and ∼37% of the 200 had previously been identified as chemical-, radiation- or UV-sensitive (Chan *et al*., 2000; Desmoucelles *et al*., 2002; Fleming *et al*., 2002; Giaever *et al*., 2002; Chang *et al*., 2002; Hanway *et al*., 2002; Bennett *et al*., 2001). Notably, we saw a strong correlation with slow-growing strains and strains that were sensitive to oxidative stress, with ∼80% of the 200 poorest growing strains sensitive to $H_2O_2$ and/or menadione (data not shown). This result may reflect the relationship between peroxide sensitivity and the growth state of the strains, as exponentially-growing cells are more sensitive than stationary-phase cells (Jamieson, 1992).

When the strains were ordered by chromosomal position of the deleted gene, we observed that some adjacent strains (∼75 pairs) showed similar chemical sensitivities. Of these, 30 pairs consisted of one strain with a deletion in a hypothetical open reading frame (ORF) that was considered unlikely by synteny analysis (Kellis *et al*., 2003) and that overlapped or was adjacent to a known or likely ORF. The chemical sensitivity due to deletion of the hypothetical ORF can most likely be attributed to full or partial deletion of the known ORF or its regulatory region, and so these strains with unlikely ORFs were eliminated from the dataset (Supplemental Table 1a). An additional 16 strains (Supplemental Table 1b) also had deletions in unlikely ORFs (Kellis *et al*., 2003) but were not removed, as they did not have a neighboring strain with a similar sensitivity pattern.

659 strains (Supplemental Table 2) showed sensitivity to at least one chemical, as indicated by a median growth ratio of >1.5 (Figure 1). 163 strains showed sensitivity to at least two chemicals, excluding those sensitive only to $H_2O_2$ and menadione. These multichemical-sensitive (MCS) strains fell into several classes, and included deletions in chromatin, transcription, cell structure and vacuolar functions. Additional strains will likely be characterized as MCS upon screening with other chemicals. When we compared the 659 sensitive strains with other annotated deletion phenotypes, we observed that ∼200 overlap with strains previously characterized as having a growth defect with chemicals (Chan *et al*., 2000; Desmoucelles *et al*., 2002; Fleming *et al*., 2002; Giaever *et al*., 2002; Chang *et al*., 2002), or radiation (Bennett *et al*., 2001) or on a particular medium (Giaever *et al*., 2002; Steinmetz *et al*., 2002) (Figure 2). Much of the overlap is due to $H_2O_2$-sensitive strains that have a growth defect on non-fermentable carbon sources (Steinmetz *et al*., 2002).

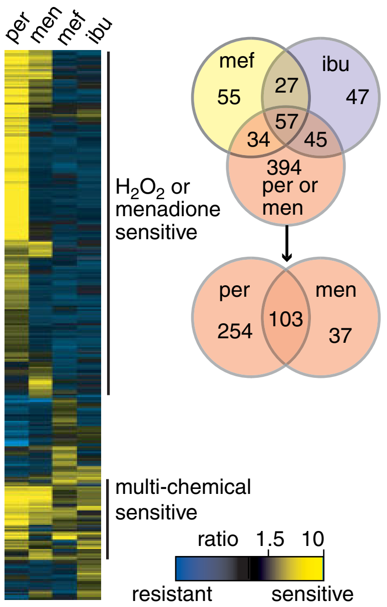

**Figure 1.** Chemical-sensitive deletion strains. Hierarchical clustering of 659 chemical-sensitive strains with growth ratios >1.5 shown in yellow. Columns show sensitivity to hydrogen peroxide (per), menadione (men), mefloquine (mef) and ibuprofen (ibu). Strains were hierarchically clustered using Cluster and visualized using TreeView (**http://rana.lbl.gov/EisenSoftware.htm**). Venn diagrams of sensitive strains show specific sensitivity to each chemical

Copyright © 2004 John Wiley & Sons, Ltd. 

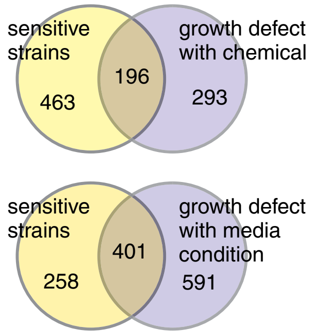

**Figure 2.** Correlation with other deletion strain studies. The first Venn diagram shows correlation of our sensitive strains (yellow circle) with screens with nystatin (Giaever *et al.*, 2002), MMS (Chang *et al.*, 2002), PS-341 (Fleming *et al.*, 2002), MPA (Desmoucelles *et al.*, 2002), rapamycin (Chan *et al.*, 2000) or $\gamma$-irradiation (Bennett *et al.*, 2001) (purple circle). The second diagram shows correlation with strains showing growth defects on fermentable or non-fermentable carbon sources, in complete-synthetic media, at pH 8, in 1 M NaCl, or in various drop-out media (Giaever *et al.*, 2002; Steinmetz *et al.*, 2002) (purple)

### Strains with sensitivity to oxidative stress

Cells are exposed to reactive oxygen species (ROS) by free radical-generating compounds and as a normal by-product of aerobic respiration, which generates ROS in the mitochondria. Without neutralization, these ROS can extensively damage proteins, lipids and nucleic acids. To prevent this, aerobic organisms have evolved extensive primary and secondary defences, including antioxidant enzymes that neutralize ROS and mechanisms for repairing DNA and eliminating damaged molecules.

We identified 394 strains, upon removal of MCS strains, that were significantly sensitive to the oxidants $H_2O_2$ or menadione (Figure 3). Although both $H_2O_2$ and menadione generate ROS, they act differently: $H_2O_2$ can be reduced by metals via the Fenton reaction to form hydroxyl radicals, whereas menadione can form superoxide, $H_2O_2$ and semiquinone radicals. The different effects of these breakdown products are reflected by the sensitivity profiles, with 103 strains sensitive to both oxidants, 254 specific to $H_2O_2$ and 37 to menadione. The *ctr1, lys7* and *sod1* strains were extremely sensitive to menadione, a superoxide generator, but not to $H_2O_2$ (Figure 3). Ctr1, a plasma-membrane copper transporter, transports copper to Lys7, which shuttles copper to the Cu/Zn superoxide dismutase, Sod1, which neutralizes highly reactive superoxide ions.

Many strains deficient in known genes involved in protection from oxidative stress were sensitive to $H_2O_2$ and/or menadione. The strains deleted for *SKN7* and *YAP1*, encoding transcription factors that initiate a global response to oxidative stress, were two of the most sensitive to $H_2O_2$. Strains deficient in antioxidant functions, including thioredoxin peroxidase (*tsa1*), glutathione peroxidase (*hyr1*), glutaredoxin (*grx5*), cytochrome C peroxidase (*ccp1*) and thioredoxin II (*trx2*), were also sensitive to $H_2O_2$ and/or menadione. Both the glutathione and thioredoxin antioxidant pathways require NADPH, generated by the pentose phosphate pathway, for their reducing power. Deletions affecting enzymes of this pathway, including ribulose-phosphate 3-epimerase (Rpe1), glucose-6-phosphate 1-dehydrogenase (Zwf1) and transketolase (Tkl1), have been observed to be sensitive to $H_2O_2$ (Juhnke *et al.*, 1996) and were strongly sensitive to oxidants in our screens. Thirteen oxidant-sensitive strains contain deletions in DNA repair genes, including those encoding the apurinic/apyrimidinic (AP) endonuclease *APN2* and the DNA glycosylase/AP lyase *NTG1*, and genes involved in the *RAD52* pathway of double-strand break repair (*RAD52, RAD50, MRE11* and *XRS2*).

The largest group of strains with specific sensitivity to $H_2O_2$ contains deletions in genes for mitochondrial functions, including protein synthesis, respiration and mitochondrial genome maintenance. Although the mitochondria generate most of the endogenous ROS in the cell through the electron transport chain, loss of mitochondrial function is associated with sensitivity to oxidative stress (Grant *et al.*, 1997). It has been speculated that a process for neutralizing ROS or repairing oxidative damage exists that requires energy generated by the mitochondria (Grant *et al.*, 1997).

Seventy-seven strains sensitive to $H_2O_2$ or menadione contained a deletion in an uncharacterized gene, as annotated in the *Saccharomyces* Genome Database. The 21 strains most sensitive to $H_2O_2$ included nine with deletions in genes for uncharacterized proteins at the time we initially analysed the data. Recently, two of these nine were characterized with important roles in mediating oxidative stress responses: YBR216C (YBP1), which interacts with Yap1 and is required for the oxidative stress response to peroxides (Veal *et al.*,

Copyright © 2004 John Wiley & Sons, Ltd.

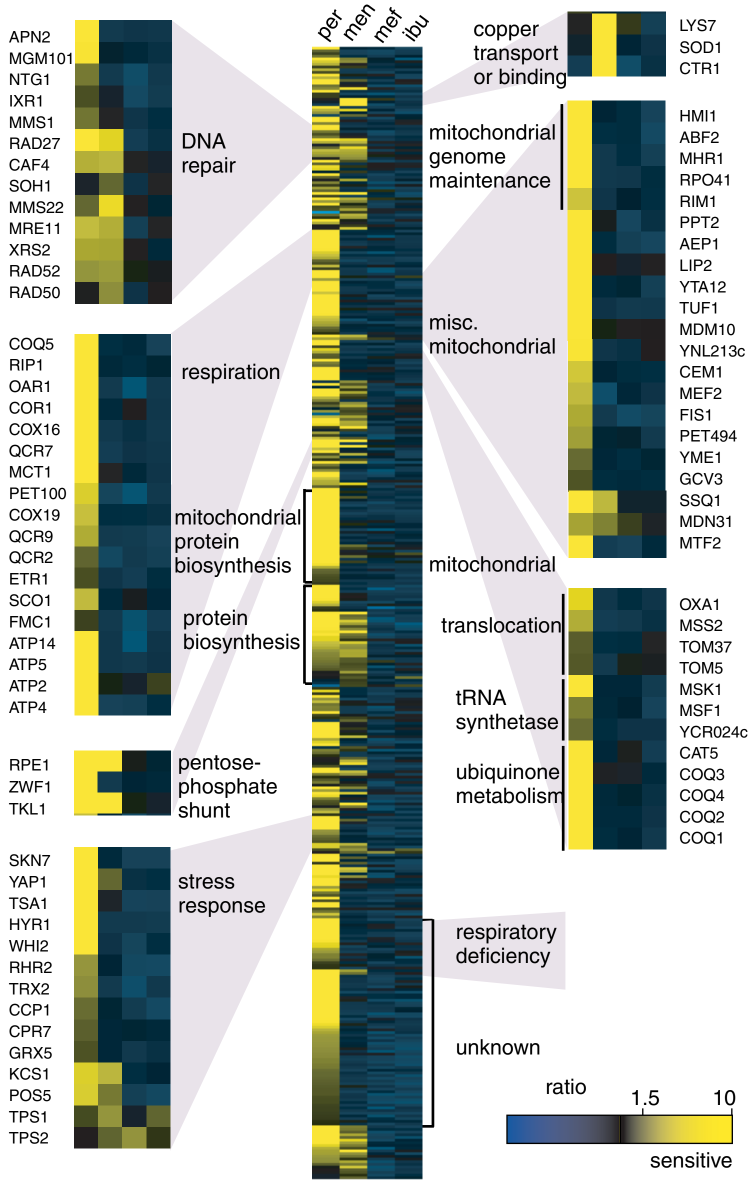

**Figure 3.** Strains sensitive to $H_2O_2$ and/or menadione. Strains were hierarchically clustered using Cluster, then grouped according to process and visualized using TreeView. Sensitive strains with a ratio >1.5 are indicated in yellow. Columns represent $H_2O_2$ (per), menadione (men), mefloquine (mef) and ibuprofen (ibu) profiles. Shown at left and right sides are expanded views of profiles, organized by functional category

2003), and YKL086W (Srx1), a novel sulphiredoxin (Biteau *et al.*, 2003). Another of the nine proteins (YPR116W) is localized to the mitochondria (Kumar *et al.*, 2002), two (YDL091C, Rtn2) have their genes transcriptionally upregulated in response to $H_2O_2$ (Causton *et al.*, 2001), and three strains (*ydr065w, ydl114w, yhr168w*) have respiratory deficiencies, suggesting a mitochondrial association (Steinmetz *et al.*, 2002).

### Ibuprofen- and mefloquine-sensitive deletion strains

Ibuprofen, an antiinflammatory, and mefloquine, an antimalarial drug, are widely used in humans but

Copyright © 2004 John Wiley & Sons, Ltd. 

are toxic to yeast. Ibuprofen inhibits the cyclooxygenase proteins in humans, which are not present in yeast, while mefloquine has an unknown mechanism of action. To examine the cellular processes targeted by these compounds with unknown mechanisms of action, we screened the deletion set with ibuprofen and mefloquine and identified strains that are specifically sensitive to each drug.

In the screen with ibuprofen, we identified 176 sensitive strains. Upon removal of MCS strains, 47 strains were specifically sensitive and of these, 28 could be placed into four functional categories (Figure 4), indicating the compound targets a specific set of cellular processes. Noticeably, deletion of any of seven genes involved in biosynthesis of tryptophan resulted in strong ibuprofen sensitivity. Since addition of tryptophan to the media increases resistance of yeast to both FK506 and isoflurane (Heitman *et al.*, 1993; Palmer *et al.*, 2002), this amino acid may have a general role in chemical resistance. An additional 10 strains specifically sensitive to ibuprofen are deleted for genes encoding transporters or regulators of transporters. Three strains, *alf1, gim5* and *yke2*, contain deletions in genes needed for tubulin folding, suggesting a cytoskeletal association. Nine strains, including *cog1* and *cog5* of the Golgi transport complex, contain deletions in genes involved in protein processing, transport through the ER and Golgi or vacuolar transport. Other *cog* strains showed sensitivity to ibuprofen only, but at levels below the 1.5-fold cut-off, including *cog6* (ratio 1.42) and *cog7* (ratio 1.4). Studies on *Candida albicans* suggest that ibuprofen may cause significant damage to the plasma membrane (Pina-Vaz *et al.*, 2000). Deletions in genes involved in membrane protein processing may slow repair of the plasma membrane, resulting in increased lethality.

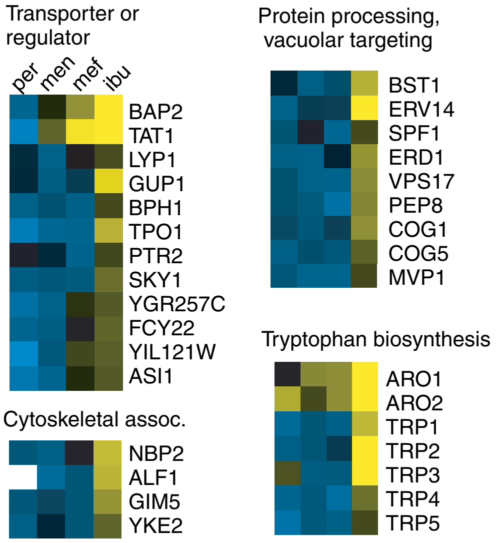

**Figure 4.** Ibuprofen-sensitive strains; 28 strains specifically sensitive to ibuprofen, as well as four additional strains with multiple chemical sensitivities and strong ibuprofen-sensitive phenotypes, are shown

In the mefloquine screen, we found 173 sensitive strains, with 55 specifically sensitive upon removal of MCS strains. These show no clear pattern, and include deletions in genes associated with a range of activities. The deletion for the gene *STI1*, which causes mefloquine resistance when overexpressed and encodes an Hsp90 cochaperone (Delling *et al.*, 1998), was sensitive to mefloquine only. Strains deleted for genes in the MAP-kinase pathway involved in maintenance of cell integrity, including *bck1, slt2* and *rlm1,* showed a strong phenotype with mefloquine, but at least two of these strains are sensitive to other agents as well (Chan *et al.*, 2000; Chang *et al.*, 2002; SenGupta *et al.*, 2003; Wantanabe *et al.*, 1985).

### Correlation with other genome-wide studies

Yeast analyses have generated a vast amount of information regarding protein interactions, protein localizations, gene transcription and gene deletion phenotypes. We used the program Osprey (Breitkreutz *et al.*, 2003) to combine the chemical sensitivity data with data from large-scale two-hybrid screens (Ito *et al.*, 2001; Uetz *et al.*, 2000); 82 interacting pairs were identified among the proteins corresponding to the 659 sensitive deletion strains. Of these, 38 pairs had corresponding deletion strains with similar sensitivity profiles. These included two strongly $H_2O_2$-sensitive strains, corresponding to the proteins YBR216C and Yap1 (Figure 5a), and five ibuprofen-sensitive strains, including three with deletions in genes with unknown functions (Figure 5b). The phenotype data and association of YBR216C with Yap1, the oxidative stress-induced transcription factor, strongly implicated YBR216C as a novel component of the oxidative stress response, and this protein has recently been characterized with a role in the hydrogen peroxide response pathway (Veal *et al.*, 2003).

Copyright © 2004 John Wiley & Sons, Ltd. 

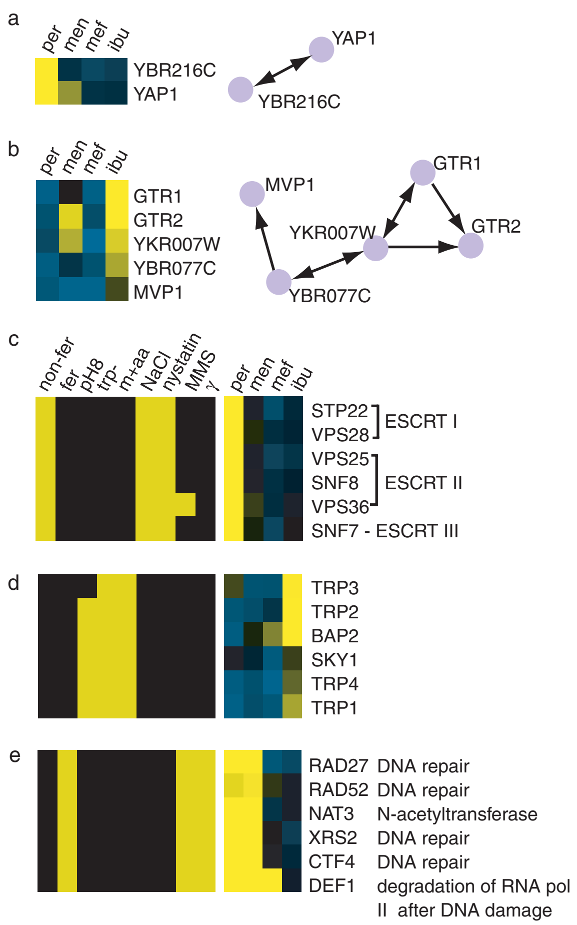

**Figure 5.** Correlation with genome-wide data. (a) Profiles of *yap1* and *ybr216c* deletion strains, with two-hybrid interaction map of corresponding proteins shown. Interactions were identified using Osprey (**http://biodata.mshri.on.ca:80/osprey/servlet/Index**). (b) Gtr1/Gtr2 cluster. Profiles of deletion strains are shown next to interaction map. (c–e) Three clusters of deletion strains with similar phenotypes. Headings indicate experiments testing for growth defect on non-fermentable (non-fer) or fermentable carbon source (fer) (Steinmetz *et al.*, 2002); media of pH 8 (pH8), tryptophan drop-out media (trp$^-$), minimal medium supplemented with histidine, leucine, uracil (m + aa), 1 M NaCl (NaCl), nystatin (Giaever *et al.*, 2002), methyl methanesulphonate (MMS) (Chang *et al.*, 2002) or $\gamma$-irradiation ($\gamma$) (Bennett *et al.*, 2001). Experiments were assigned a value of '1' or '0' indicating growth defect, and hierarchically clustered with peroxide (per), menadione (men), mefloquine (mef) and ibuprofen (ibu) data using Cluster

We used hierarchical clustering to compare our deletion profiles with annotated deletion phenotypes (Giaever *et al.*, 2002; Chang *et al.*, 2002; Bennett *et al.*, 2001; Steinmetz *et al.*, 2002). One group of strains with sensitivity to NaCl and nystatin and poor growth on non-fermentable carbon sources clustered with $H_2O_2$-sensitive strains (Figure 5c). These strains all have deletions affecting proteins of the ESCRT pathway, which is involved in sorting of proteins in the late endosome into multivesicular bodies for degradation (Lemmon and Traub, 2000). Another group of six strains had phenotypes that clustered with ibuprofen-sensitive strains (Figure 5d). Four of the six encode proteins involved in tryptophan biosynthesis, one (Bap2) is an amino acid permease, and the last (Sky1) is a protein kinase. Based on these associations, Sky1 may function in regulating amino acid uptake. Six other strains (Figure 5e) had phenotypes that clustered with strains sensitive to both $H_2O_2$ and menadione. Of these, five encode proteins involved in response to DNA damage.

### Concluding remarks

The generation of a quantitative set of chemical sensitivity profiles for the MAT$\alpha$ yeast deletion collection allows us to categorize genes with similar deletion phenotypes into functional groups on a level not possible with qualitative measurements. By combining quantitative measurements with multiple repetitions (typically six screens for each chemical), we were able to identify subtle but reproducible growth defects. The raw data can be reanalysed, e.g. using cut-offs other than the 1.5 ratio used in this analysis, or using a difference measurement rather than a ratio, which would remove slow-growing strains.

Our screens were carried out in the BY4742 *MAT$\alpha$* set of deletion strains. Analysis of the BY4743 diploid deletion strains has indicated widespread (~8%) aneuploidy (Hughes *et al.*, 2000), and small mutations are also likely to be present throughout the strains. Additionally, we have found differences between the BY4742 *MAT$\alpha$* strains and the BY4741 *MAT***a** strains (unpublished data). As we screened only haploid strains, we were able to characterize only ~4800 strains out of over 6000. Further studies could include characterization of the essential gene deletions using the set of heterozygous diploid strains, as well as screening of the *MAT***a** strains.

Copyright © 2004 John Wiley & Sons, Ltd. 

By correlating our data from multiple chemical screens, we are able to better understand the specific functions perturbed by each chemical (such as DNA damage with oxidative stress), as well as the sources of multiple chemical sensitivity. Many strains we identified as MCS had been previously identified in screens with other chemicals, but their sensitivity had been attributed to direct effects of the chemical on its target. We also correlated our data with other genomic screens, such as protein interaction studies, finding that, as with other genome-wide data, the value of this chemical sensitivity data set is enhanced greatly by its correlation to results from other large- and small-scale studies.

### Acknowledgements

We thank Eric Phizicky, Dan Gottschling, Victoria Brown-Kennerly and Tony Hazbun for helpful comments on the manuscript, and Ainslie Parsons and Charlie Boone for sharing their unpublished data. C.L.T. was supported by NRSA Fellowship 1F32GM20532-01. S.F. is an investigator of the Howard Hughes Medical Institute.

### References

Begley TJ, Rosenbach AS, Ideker T, Samson LD. 2002. Damage recovery pathways in *Saccharomyces cerevisiae* revealed by genomic phenotyping and interactome mapping. *Mol Cancer Res* **1**: 103–112.

Bennett CB, Lewis LK, Karthikeyan G, *et al.* 2001. Genes required for ionizing radiation resistance in yeast. *Nature Genet* **29**: 426–434.

Biteau B, Labarre J, Toledano MB. 2003. ATP-dependent reduction of cysteine-sulphinic acid by *S. cerevisiae* sulphiredoxin. *Nature* **425**: 980–984.

Blackburn A, Avery S. 2003. Genome-wide screening of *Saccharomyces cerevisiae* to identify genes required for antibiotic insusceptibility of eukaryotes. *Antimicrob Agents Chemother* **47**: 676–681.

Breitkreutz BJ, Stark C, Tyers M. 2003. Osprey: a network visualization system. *Genome Biol* **4**: R22.

Causton HC, Ren B, Koh SS, *et al.* 2001. Remodeling of yeast genome expression in response to environmental changes. *Mol Biol Cell* **12**: 323–337.

Chan TF, Carvalho J, Riles L, Zheng XF. 2000. A chemical genomics approach toward understanding the global functions of the target of rapamycin protein (TOR). *Proc Natl Acad Sci USA* **97**: 13 227–13 232.

Chang M, Bellaoui M, Boone C, Brown GW. 2002. A genome-wide screen for methyl methanesulfonate-sensitive mutants reveals genes required for S phase progression in the presence of DNA damage. *Proc Natl Acad Sci USA* **99**: 16 934–16 939.

Delling U, Raymond M, Schurr E. 1998. Identification of *Saccharomyces cerevisiae* genes conferring resistance to quinoline ring-containing antimalarial drugs. *Antimicrob Agents Chemother* **42**: 1034–1041.

Desmoucelles C, Pinson B, Saint-Marc C, Daignan-Fornier B. 2002. Screening the yeast 'disruptome' for mutants affecting resistance to the immunosuppressive drug, mycophenolic acid. *J Biol Chem* **277**: 27 036–27 044.

Fleming JA, Lightcap ES, Sadis S, *et al.* 2002. Complementary whole-genome technologies reveal the cellular response to proteasome inhibition by PS-341. *Proc Natl Acad Sci USA* **99**: 1461–1466.

Giaever G, Chu AM, Ni L, *et al.* 2002. Functional profiling of the *Saccharomyces cerevisiae* genome. *Nature* **418**: 387–391.

Grant CM, MacIver FH, Dawes IW. 1997. Mitochondrial function is required for resistance to oxidative stress in the yeast *Saccharomyces cerevisiae*. *FEBS Lett* **410**: 219–222.

Hanway D, Chin JK, Xia G, *et al.* 2002. Previously uncharacterized genes in the UV- and MMS-induced DNA damage response in yeast. *Proc Natl. Acad Sci USA* **99**: 10 605–10 610.

Heitman J, Koller A, Kunz J, *et al.* 1993. The immunosuppressant FK506 inhibits amino acid import in *Saccharomyces cerevisiae*. *Mol Cell Biol* **13**: 5010–5019.

Higgins VJ, Alic N, Thorpe GW, *et al.* 2002. Phenotypic analysis of gene deletant strains for sensitivity to oxidative stress. *Yeast* **19**: 203–214.

Hughes TR, Roberts CJ, Dai H, *et al.* 2000. Widespread aneuploidy revealed by DNA microarray expression profiling. *Nature Genet* **25**: 333–337.

Ito T, Chiba T, Ozawa R, *et al.* 2001. A comprehensive two-hybrid analysis to explore the yeast protein interactome. *Proc Natl Acad Sci USA* **98**: 4569–4574.

Jamieson DJ. 1992. *Saccharomyces cerevisiae* has distinct adaptive responses to both hydrogen peroxide and menadione. *J Bacteriol* **174**: 6678–6681.

Juhnke H, Krems B, Kotter P, Entian KD. 1996. Mutants that show increased sensitivity to hydrogen peroxide reveal an important role for the pentose phosphate pathway in protection of yeast against oxidative stress. *Mol Gen Genet* **252**: 456–464.

Kellis M, Patterson N, Endrizzi M, Birren B, Lander ES. 2003. Sequencing and comparison of yeast species to identify genes and regulatory elements. *Nature* **423**: 233–234.

Kumar A, Agarwal S, Heyman JA, *et al.* 2002. Subcellular localization of the yeast proteome. *Genes Dev* **16**: 707–719.

Lemmon SK, Traub LM. 2000. Sorting in the endosomal system in yeast and animal cells. *Curr Opin Cell Biol* **12**: 457–466.

Palmer LK, Wolfe D, Keeley JL, Keil RL. 2002. Volatile anaesthetics affect nutrient availability in yeast. *Genetics* **161**: 563–574.

Pina-Vaz C, Sansonetty F, Rodrigues AG, *et al.* 2000. Antifungal activity of ibuprofen alone and in combination with fluconazole against *Candida* species. *J Med Microbiol* **49**: 831–840.

Sen Gupta S, Van-Kuhe T, Beaudry V, *et al.* 2003. Antifungal activity of amiodarone is mediated by disruption of calcium homeostasis. *J Biol Chem* **278**: 28 831–28 839.

Steinmetz LM, Scharfe C, Deutschbauer AM, *et al.* 2002. Systematic screen for human disease genes in yeast. *Nature Genet* **31**: 400–404.

Uetz P, Giot L, Cagney G, *et al.* 2000. A comprehensive analysis of protein–protein interactions in *Saccharomyces cerevisiae*. *Nature* **403**: 623–627.

Copyright © 2004 John Wiley & Sons, Ltd. 

Veal EA, Ross SJ, Malakasi P, Peacock E, Morgan BA. 2003. Ybp1 is required for the hydrogen peroxide-induced oxidation of the Yap1 transcription factor. *J Biol Chem* **15**: 30 896–30 904.
Wantanabe Y, Irie K, Matsumoto K. 1995. Yeast RLM1 encodes a serum response factor-like protein that may function downstream of the Mpk1 (Slt2) mitogen-activated protein kinase pathway. *Mol Cell Biol* **15**: 5740–5749.
Winzeler EA, Shoemaker DD, Astromoff A, *et al*. 1999. Functional characterization of the *S. cerevisiae* genome by gene deletion and parallel analysis. *Science* **285**: 901–906.
Zewail A, Xing Y, Lin L, *et al*. 2003. Novel functions of the phosphatidylinositol metabolic pathway discovered by a chemical genomics screen with wortmannin. *Proc Natl Acad Sci USA* **100**: 3345–3350.

Copyright © 2004 John Wiley & Sons, Ltd. 
